# Analysis of Serum Biochemical Indexes, Egg Quality, and Liver Transcriptome in Laying Hens Fed Diets Supplemented with *Gynostemma pentaphyllum* Powder

**DOI:** 10.3390/genes12121942

**Published:** 2021-11-30

**Authors:** Tao Li, Shuya Zhang, Jiqiao Zhang, Yiping Song, Xiuyu Bao, Fengwen Xu, Jianqin Zhang

**Affiliations:** 1 College of Animal Science and Technology, Northwest A&F University, Xianyang 712100, China; 18392459744@nwafu.edu.cn (T.L.); shuyazhang2020@163.com (S.Z.); m15667895160@163.com (J.Z.); 18735431454@nwafu.edu.cn (Y.S.); xiuyu@nwafu.edu.cn (X.B.); 2 Animal Husbandry and Veterinary Center of Ankang City, Ankang 725000, China; xufengwenshan@163.com

**Keywords:** *Gynostemma pentaphyllum*, laying hens, total cholesterol, serum, transcriptome

## Abstract

*Gynostemma pentaphyllum* (*GP*), known as “southern ginseng”, can reduce the blood pressure and blood lipid levels. In this study, 300 layer chicks of one day old were divided randomly into three groups (control group (base diet), high addition group (base diet with 1% *GP*), and low addition group (base diet with 0.5% *GP*)). After 29 weeks, the growth performance, egg quality, and serum index were determined. Additionally, liver mRNA was identified using RNA-seq to investigate the molecular mechanisms. The results indicated that the serum total cholesterol and triglycerides decreased significantly in the *GP* addition group. The addition of *GP* increased the egg weight, Haugh unit and redness (a*) of the egg yolk color, and reduced the yolk cholesterol concentration. Moreover, 95 differentially expressed genes (DEGs) were screened between the control and *GP* addition group. GO and the KEGG analysis showed that the PPAR pathway was significantly enriched. Five fatty acid metabolism-related genes (*FABP3*, *CYP7A1*, *ANKRD22*, *SCD1*, and *PCK1*) were validated by qRT-PCR analysis, which confirmed the tendency of the expression. These DEGs in the PPAR pathway may be the key factors of *GP* affecting fatty acid metabolism. These results may provide a theoretical basis for further research and new insights into *GP* as a feed additive.

## 1. Introduction

*Gynostemma pentaphyllum* (*GP*), also called five-leaf ginseng, sweet tea vine, and gospel herb, is a dioecious, herbaceous climbing vine and a member of the Cucurbitaceae family (cucumber or gourd family). *GP* is widely distributed in South and East Asia and New Guinea [1] and grows in shady places in the mountains at altitudes of 300–3200 m above sea level. A Chinese plant that is a precious source of saponins, *GP* does not only contain eight kinds of gypenosides, which have the same chemical structures as ginsenosides Rb1, Rb3, Rc, Rd, F2, Rg3, malonyl-Rb1, and malonyl-Rd, but also more than 169 other gypenosides [2]. Gypenosides have pharmacological properties, such as anticancer [3], antioxidative [4], anti-inflammatory [5], antistress [1], and hypoglycemic properties [6]. The main components of *GP* other than saponins include polysaccharides, flavonoids, amino acids [7,8], vitamins, and some essential trace elements [9]. These components are related to the pharmacological properties of *GP*, which include the strengthening of the immune system and lowering the blood sugar and blood lipid levels, as well as antitumor and anticancer properties [10]. 

Cholesterol, a vital constituent of animal cell membranes, serves as a precursor to various biomolecules (steroids hormones, bile acids, and cholecalciferol) and plays an important role in basic cell life activities. The content of serum cholesterol is one of the metrics that characterizes cardiovascular health. Under normal circumstances, the cholesterol synthesized by the body in the liver and ingested from food will be transformed into steroid hormones or components of the cell membrane, and the concentration of cholesterol in the blood will be kept constant. When severe liver lesions occur, cholesterol concentrations decrease. Excessive cholesterol intake will cause hypercholesterolemia, which will lead to coronary atherosclerotic heart disease and other diseases. Chicken eggs are highly nutritious foods; however, their high cholesterol levels (contained in the yolk) are of concern, as there exists a relationship between dietary cholesterol and atherosclerotic cardiovascular risk [11]. Therefore, a low cholesterol intake is recommended [12]. If the cholesterol level of eggs can be reduced by feed additives, consumers’ concerns about egg yolk cholesterol can be effectively reduced.

In addition, as the current trend is to reduce and replace antibiotics, the research and popularization of plant-derived feed additives, such as those of Chinese herbal medicine origin, play an essential role in the development of feed additives [13]. For about 50 years, antibiotics have been used as growth promoters in livestock production [14]. However, long-term addition or excessive addition cause antibiotics to not be excreted from the body in time [15]. Antibiotic residues in animal products can affect the product quality, lead to drug resistance [16], and even pollute the environment to a certain extent through feces excretion [17]. Solutions to the problems caused by antibiotics, such as product residues [18], environmental pollution [13], and adverse effects on animal products, have increasingly become the focus of the development of the feed additive industry [19]. 

As a high-quality herbal medicine, *Gynostemma pentaphyllum* (*GP*) has good application prospects as a feed additive. Previous studies have shown that gypenoside liposomes can significantly enhance chicken lymphocyte proliferation, increase antibody titers, and promote cytokine secretion [20]. In addition, *GP* can inhibit the apoptosis of duck embryo hepatocytes induced by duck hepatitis A virus type 1 (*DHAV-1*), thereby exerting hepatoprotective effects [21], and exerts an anti-bovine viral diarrhea virus (BVDV) infection role by inhibiting viral attachment and internalization and selectively purging virally infected cells [22]. However, there are few studies on the effects of *GP* on hen performance and lipid metabolism. Based on the functional characteristics of Gynostemma pentaphyllum and complying with the development trend of the poultry breeding industry, in this study, we hypothesized that: (1) the addition of *GP* to the routine diet of laying hens could improve their growth performance, (2) a *GP* addition would reduce the cholesterol levels in the serum and eggs of laying hens, and (3) the addition of *GP* could alter the expression of regulated genes in the liver. Therefore, *GP* powder was added to the basal diet of 0–29-week-old laying hens. The effect of *GP* powder on the serum biochemical indexes, egg quality, and liver transcriptomes of laying hens was investigated. This study lays a theoretical foundation for the application of *GP* as a feed additive and provides new insights into the development of Chinese herbal medicine additives.

## 2. Materials and Methods

### 2.1. Ethics Statement

Animal care, slaughter, and experimental procedures were approved by the Institutional Animal Care and Institutional Ethic Committee of Northwest A&F University (ethic code: #0217/2018).

### 2.2. Preparation of Gynostemma Pentaphyllum

The fresh stems and leaves of *GP* used in this study were collected from the local market (Ankang, Shaanxi, China), washed thoroughly with distilled water, and dried at room temperature for 2 to 3 days. Next, the dried stems and leaves were ground into powder using a pulverizing machine (WN-200, Langxu, Guangdong, China) and sieved through a 200–300-mesh sieve. Finally, the prepared powder was preserved (sealed condition, stored at 4 °C and no kind of preservative substances was used) until it was added to the base diet as an additive. The nutritional value and active compounds of *Gynostemma pentaphyllum* are shown in Table 1. 

### 2.3. Animals and Experimental Design

A total of 300 one-day-old Jing Fen No. 2 layers with average body weights (40 ± 3 g) were purchased from the Xingsheng Livestock and Poultry Breeding Co., Ltd. (Ankang, China). The laying hens were randomly divided into two experimental groups (a high-addition (HA) group and low-addition (LA) group) and a control group, with five replicates per group. *GP* powder was added to the basal diet of the HA and LA groups (1% and 0.5%, respectively), while the control group was fed a basal diet. GP powder was mixed with the base diet before feeding the chickens. The entire experimental period lasted 0–29 weeks. The basic feed and feeding management mode, including cage-rearing, temperature (20 °C–26 °C), humidity (65%), light (the light intensity and time were different in different growth periods), and ventilation, strictly followed the conventional feeding methods for laying hens (the laying hens manual guidance of China, NY/T 33-2004). The composition and nutrient levels of the basal diet are shown in Table 2; this was formulated to meet the nutrient requirements of laying hens [24]. During the experimental period, the laying hens were allowed ad libitum access to feed and water. 

### 2.4. Analysis of Body Weight and Feed Intake

At 2–20 weeks, the body weight (BW) and feed intake of the laying hens per replicate were measured and recorded every two weeks using an electric platform scale (TSC-E, Suowo, Guangdong, China). The average daily BW gain, average daily feed intake (ADFI), and feed conversion ratio (FCR) were calculated for the experimental periods from 2 to 20 weeks.

### 2.5. Determination of Egg Quality and Egg Physical Index 

At 29 weeks of age, 20 fresh eggs were randomly selected from each group (4 eggs from each replicate subgroup). The Haugh unit (HU), egg yolk proportion (EYP), and eggshell thickness (EST) were calculated, and the egg yolk color (EYC), egg shape index (ESI), and egg weight (EW) were measured (Robotmation Egg Analyzer, EMT-5200, Tokyo, Japan; vernier caliper, Airuize, CJW888, Shandong, China; and Roche colorimetric fan, Roche, robotmation, Basel, Switzerland) [25]. Meanwhile, 15 eggs were randomly chosen from each group, and the cholesterol concentration in the egg yolk was determined using an ELISA kit (MEIMIAN, Jiangsu, China) [26].

### 2.6. Serum Biochemical Analysis 

At the age of 29 weeks (the peak laying period), 15 hens (three hens per replication) were randomly selected from each group to collect blood from the inferior wing vein. The blood samples were centrifuged at 3000 rpm for 15 min, and the serum was collected to detect the total cholesterol (TC), total protein (TP), and triglycerides (TG) contents. 

The TP content was determined using the biuret method following the manufacturer’s instructions (total protein quantitative assay kit, Jiancheng, Nanjing, China). The TC and TG contents were detected by enzyme colorimetry using a Triglyceride Detection Kit (GPO-PAP, Jiancheng, Nanjing, China) and Cholesterol Detection Kit (CHOD-PAP, Jiancheng, Nanjing, China), respectively, according to the manufacturers’ instructions [27].

### 2.7. RNA Sequencing of Liver Tissue

The HA and control groups were selected as study objects according to the serum biochemical index and egg quality analysis results. At the age of 29 weeks (the peak laying period), seven healthy hens were randomly selected from each group and slaughtered, while the liver samples were collected quickly after. Fresh tissues were immediately frozen in liquid nitrogen and stored at −80 °C until further use.

#### 2.7.1. RNA Isolation and RNA Sequencing Library Construction

Three liver samples from each group were randomly selected for RNA sequencing. Total RNA was isolated from the liver using a TRIzol reagent (Invitrogen, Carlsbad, CA, USA) following the manufacturer’s instructions [28]. The optical density (OD) and total RNA yield were measured using NanoDrop 2000 (Thermo Scientific, Wilmington, DE, USA). The integrity was evaluated using Agilent Bioanalyzer 2100 (Agilent Technologies, Santa Clara, CA, USA) [29]. Samples with RNA integrity number (RIN) values > 8.0 and 1.9 < OD 260/280 < 2.0 were selected for library construction.

#### 2.7.2. RNA Sequencing and Data Analysis

RNA sequencing was performed on an Illumina HiSeq 2500 platform (Illumina Inc., San Diego, CA, USA) by a commercial service provider. Low-quality reads (the base number of the quality value Q ≤ 10 accounted for more than 20% of the total reads), reads containing adapters, and reads in which the proportion of poly-N was greater than 5% were removed from the raw data. Moreover, the reads containing rRNA were removed from the raw data after being compared with the rRNA database, and the subsequent analysis was based on the filtered reads. Finally, the clean reads were compared to the Gallus gallus reference genome sequence to evaluate the overall quality of sequencing https://www.ncbi.nlm.nih.gov/genome/?term=Gallus+gallus (accessed on 20 September 2020) using HISAT2 software (v2.0.0) http://ccb.jhu.edu/software/hisat2/index.shtml (accessed on 20 September 2020).

#### 2.7.3. Differential Expression Analysis

To analyze the DEGs between the HA and control groups, either a quantitative or mRNA analysis was carried out using Kallisto software https://liorpachter.wordpress.com/tag/kallisto/ (accessed on 20 September 2020) [30]. The genes of all the samples with TPM (transcripts per million) < 1 were filtered out, and then, the DEGs between the HA group and control group were analyzed using NOIseq (v2.16.0) http://www.bioconductor.org/packages/release/bioc/html/NOISeq.html (accessed on 20 September 2020). When |log_2_FC| > 1 and the probability > 0.8, the results were considered statistically significant. 

#### 2.7.4. Analysis of GO and KEGG Functional Enrichment 

To comprehensively describe the properties and function of DEGs, GO (Gene Ontology) terms [31] and a KEGG (Kyoto Encyclopedia of Genes and Genome) pathway enrichment analysis [32] of the DEGs were implemented using the GOseq (v 1.16.2) http://www.bioconductor.org/packages/release/bioc/html/GOSeq.html (accessed on 20 September 2020) and KEGG database resources http://www.genome.jp/kegg/ (accessed on 20 September 2020), respectively. Hypergeometric tests and multiple corrections were performed in both the GO term and KEGG pathway analyses; *p* < 0.05 indicated that the DEGs were significantly enriched.

### 2.8. Quantitative Real-Time PCR (qRT-PCR) Analysis

To verify the reliability and accuracy of the sequencing results, five DEGs (*FABP3*, *CYP7A1*, *ANKRD22*, *SCD1*, and *PCK1*) that were significantly enriched in the PPAR pathway were selected for qRT-PCR verification. Seven liver samples were selected from the HA and control groups. Liver tissue was used to isolate the RNA using TRIzol Reagent (Takara Bio Inc. Otsu, Shiga, Japan). The total RNA purity and concentration were measured using Nanodrop 2000 (Thermo Fisher Scientific, Waltham, MA, USA), and the RNA integrity was checked by 1.5% agarose gel electrophoresis. The A260/A280 ratio was expected to range from 1.8 to 2.0. cDNA synthesis was performed using a two-step method offered by the PrimeScript II 1st Strand cDNA Synthesis Kit (Takara Bio Inc. Otsu, Shiga, Japan). All specific primers for qRT-PCR (Supplementary Table S1) were designed using Primer 5.0 and synthesized by Sangon Biotech (Shanghai, China). The expression levels of the selected DEGs were measured using the SYBR Primer Ex Taq™ II Kit (Takara Bio Inc. Otsu, Shiga, Japan) in a LightCycler 96 instrument qRT-PCR system (Roche Molecular Biochemicals, Mannheim, BW, Germany). The results were normalized to the expression levels of chicken β-actin using the 2^−ΔΔCt^ method for quantification. All experiments were performed in triplicate. 

### 2.9. Statistical Analysis

One-way ANOVA was used if the data were normally distributed and of equal variance. SPSS 26.0 (SPSS Inc., Chicago, IL, USA) was used for the analyses. Comparisons were performed using the least significant difference (LSD) method. The results were expressed as the mean and standard error of the mean. The statistical significance was set at *p* < 0.05, while the results were considered to be very statistically significant at *p* < 0.01. Detailed information on the statistical analyses is also provided in the captions of each figure and table.

## 3. Results

### 3.1. Growth Performance

The effects of a dietary *GP* addition on the growth performance, including the BW, ADG, ADFI, and FCR, are shown in Table 3. A dietary supplementation with *GP* had no effect on the BW, ADFI, ADG, and FCR, which indicated that *GP* did not affect the growth performance of laying hens.

### 3.2. Egg Quality and Egg Physical Index

The effect of the addition of different *GP* dosages on the physical index of the eggs during the peak laying period is shown in Table 4. The egg weights of laying hens at the peak laying period increased significantly after they were given *GP* powder (*p* = 0.0100). However, there were no significant effects on the other egg physical indices, such as the egg shape index, eggshell thickness, and the average eggshell thickness (*p* > 0.05). 

The effect of a *GP* addition on the quality of eggs during the peak laying period is shown in Table 5. Compared with the control group, the Haugh unit increased significantly when the addition of *GP* powder was 0.5% and 1.0% (*p* = 0.0421), and no significant difference was found between the LA and HA groups. The egg yolk cholesterol concentration of the HA group was significantly lower than that of the LA and control groups (*p* = 0.0078), and there was no significant difference between the LA and control groups. The redness (a*) value of the egg yolk color in the HA group was significantly higher than that in the control and LA groups (*p* = 0.0421). There was no significant difference in the values of the egg yolk proportion and in the yellowness (b*) of the egg yolk color and egg yolk lightness (L*) between the experimental groups and control group (*p* > 0.05).

### 3.3. Serum Biochemical Indexes

The effects of dietary GP addition on the serum TC, TG, and TP are shown in Table 6. Overall, the serum TG and TC contents were significantly affected by dietary GP supplementation. The TG and TC concentrations in the GP addition group were significantly lower than those in the control group (*p* = 0.0038 and *p* = 0.0065, respectively). However, there was no significant difference between the two treatment groups. Additionally, the serum TP levels tended to decrease with dietary GP supplementation, but the difference was not significant (*p* = 0.0783).

### 3.4. Overview of RNA Sequencing

The raw reads obtained by RNA sequencing were all over 8.01 × 10^7^ bp among the different samples. After filtering out low-quality reads, the clean data Q20% and Q30% were all higher than 90% in each sample, and the GC content was less than 50%. The results of the hisat2 comparison are presented in Supplementary Table S2. After the clean reads were mapped to the *Gallus gallus* reference genome, the average percentage of the total mapped and uniquely mapped reads was 95% and 62%, respectively.

### 3.5. Analysis of Differentially Expressed Genes (DEGs)

**Figure 1:**
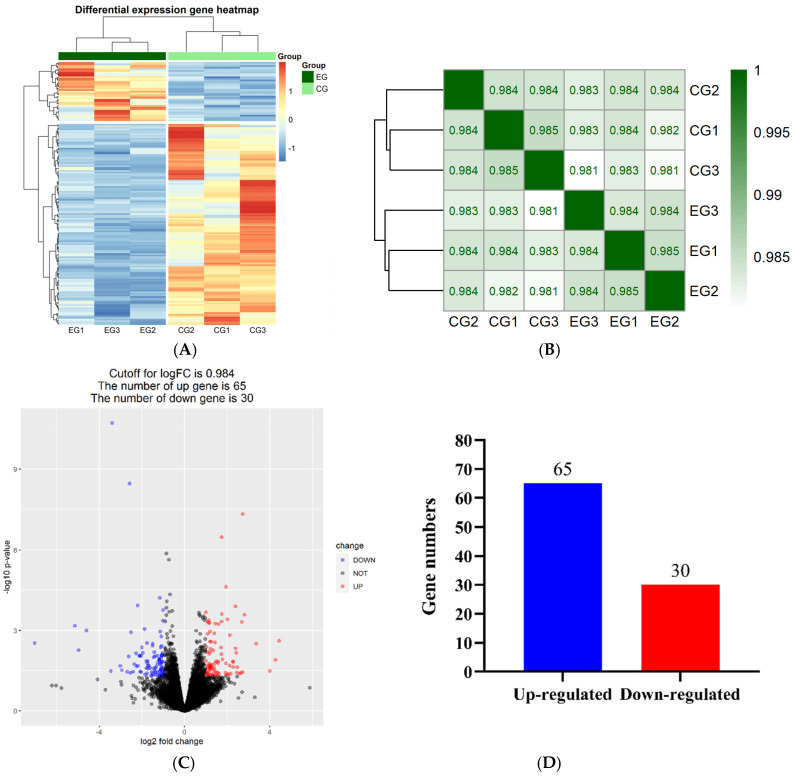
Analysis of differentially expressed genes. (**A**) Clustering analysis of DEGs between EG and CG. (**B**) Correlation matrix diagram between EG and CG; the correlation coefficient is represented by color—the darker the color of green, the higher the correlation. (**C**) Volcano plot of DEGs between EG and CG; blue and red represent downregulated and upregulated DEGs, respectively. (**D**) Statistics of the DEGs between EG and CG. EG: high-addition group, CG: control group, and DEGs: differentially expressed genes.

A heatmap (Figure 1A) was used to show the expression differences of the DEGs in the livers of the *GP* and control groups. The results of the cluster analysis revealed a distinct expression signature of the DEGs in the *GP* addition and control groups. The correlation coefficient represents the degree of similarity between the samples. A significant correlation was found between the DEGs in the *GP* addition and control groups (Figure 1B). The liver is the primary site of cholesterol biosynthesis in laying hens, and cholesterol readily transforms from the blood to the liver [33]. To identify the specific genes that participate in fatty acid metabolism in the liver, the gene expression profiles between the *GP* addition and control groups were compared in this study: 10,920 DEGs were identified in the *GP* addition groups, and 10,605 DEGs were found in the control group (Supplementary Table S3). Among them, 95 DEGs, including 65 significantly upregulated DEGs and 30 downregulated DEGs, were differentially expressed between the *GP* addition and control groups (Figure 1C,D). 

### 3.6. GO Terms and KEGG Pathway Analysis of DEGs

**Figure 2:**
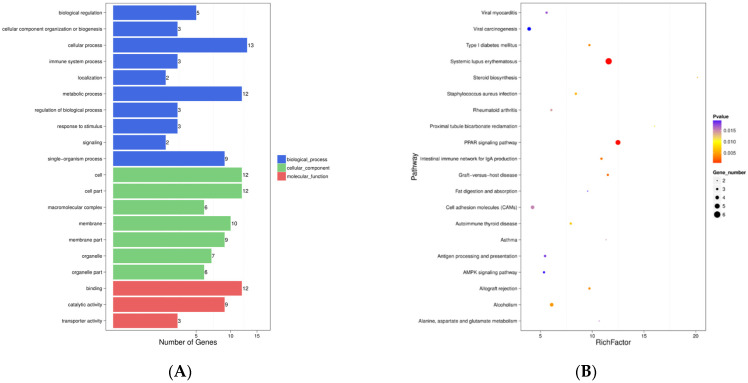
GO and KEGG enrichment analysis. (**A**) The GO terms of the enrichment analysis of the DEGs. Blue represents a biological process, green represents a cellular component, and red represents a molecular function. (**B**) The KEGG pathway enrichment analysis of the DEGs. The larger the circle area’s mean, the more differently expressed the gene number. The *p*-value represents a significant level (*p* < 0.05).

The GO analysis of the DEGs showed that the majority of the DEGs were enriched (*p* < 0.05) in biological processes (10 terms), cellular components (7 terms), and molecular functions (3 terms) (Figure 2A). Within the biological processes of the GO category, 12 genes were enriched in metabolic processes, and 13 genes were enriched in cellular processes. The KEGG pathway analysis of the DEGs revealed that the main terms, including the PPAR signaling pathway and systemic lupus erythematosus, were related to fatty acid metabolism and immunity (*p* < 0.05) (Figure 2B).

### 3.7. Verification of mRNA Expression Profiles Using qRT-PCR

**Figure 3:**
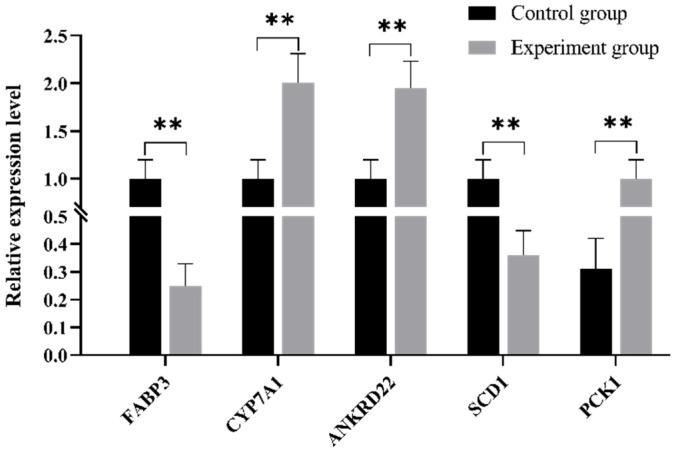
The quantitative real-time PCR validation of randomly selected differentially expressed genes (*FABP3*, *CYP7A1*, *ANKRD22*, *SCD1*, and *PCK1*). The x-axis represents differentially expressed genes, and the y-axis represents the relative expression levels of the genes. ** on the top of lines or bars indicates a significant difference (*p* < 0.01) between the high-addition group and control group.

To verify the accuracy of the RNA-seq results, five DEGs were selected for qRT-PCR verification. The results showed that the expression of *CYP7A1* and *ANKRD22* was higher in the *GP* addition group than in the control group, whereas the expression of FABP3, *SCD1*, and *PCK1* was much lower in the *GP* addition group than in the control group (Figure 3). All the expression levels of the DEGs were consistent with the RNA-Seq results, suggesting that the expression abundance of the DEGs in the RNA-seq was highly reliable.

## 4. Discussion

The relationship between food cholesterol, especially cholesterol in eggs and human cholesterol, has been studied extensively. Therefore, most egg eaters prefer to choose eggs with low cholesterol contents. *Gynostemma pentaphyllum* (*GP*), a Chinese herbal medicine, has been widely studied in the context of traditional Chinese medicine. It has anticancer, anti-inflammatory, and immunostimulatory properties [7]. In this study, the effects of *GP* on the growth performance; the physical properties of eggshells; egg quality; and the TC, TG, and TP of the chicken serum contents were studied. The results showed that *GP* significantly decreased the TC and TG levels in the serum and the TC content in egg yolk, while it increased the egg weight, egg yolk color (a), and Haugh unit; these results indicate that *GP* supplementation improved the egg yolk quality and reduced the egg cholesterol content but had no effect on the body weight, feed consumption, and egg physical index of laying hens. As an anti-inflammatory and hypolipidemic Chinese herbal medicine, *GP* may not directly affect the growth performance of laying hens. 

At present, there is no related study on the effect of *Gynostemma pentaphyllum* as a feed additive on laying hens. The results of the preliminary studies showed that *GP* lowered the cholesterol content of the serum and yolk, and a 0.5% *GP* supplementation was found to be a suitable dose to produce near-maximum decreases in the TC and TG levels. Many previous studies have shown that *GP* can suppress the plasma TG and TC levels in a rat model of colorectal cancer [34]. The saponins extracted from Gynostemma pentaphyllum can significantly improve the blood lipid level of an animal model. A dose of 50–500 mg/kg can effectively reduce the total cholesterol, triglycerides, and low-density lipoprotein cholesterol (LDL-C), respectively [35]. Megalli et al. reported that the LDL-C levels in rats were reduced by more than 30% in chronic studies of long-term *GP* supplementation [36]. Plasma LDL is the main carrier transporting endogenous cholesterol. A possible explanation for this reduction in the TG levels is that *GP* may promote TG exports to peripheral tissues from the liver or increase the fatty acid oxidation [37] and lipoprotein lipase (LPL) activity and reduce the very low-density lipoprotein (VLDL) production in the liver [38]. Although there is currently no research on *GP* as a layer feed additive, our results, which indicated that *GP* could significantly reduce TC and TG in the serum and yolk of laying hens, are consistent with the results of many studies examining *GP* as a traditional Chinese medicine. 

The liver is the main site of cholesterol synthesis, and the liver accumulates cholesterol from the blood when it synthesizes lipoproteins. To understand the influence mechanism at the molecular level, a liver RNA-seq was performed, and 95 DEGs were identified between the *GP* addition and control groups. The DEGs were mainly enriched in the PPAR signaling pathway. Studies have found that many PPAR agonists are used to treat dyslipidemia, mainly because they can reduce the triglyceride levels [39]. *GP* affects the lipid metabolism and anti-inflammation mainly through its major component, gypenoside Gyp-XLIX, which activates PPAR-α [40]. Malek et al. also confirmed that ombuin-3-O-β-D-glucopyranoside, a *GP* flavonoid, is a dual agonist of PPAR α, δ, and β and regulates the lipid metabolism of many kinds of cells [41]. An ombuin treatment led to an increased lipid uptake and gene expression in reverse cholesterol transport, fatty acid synthesis, fatty acid uptake, and fatty acid β-oxidation. Another study also found that the rat liver mRNA and protein levels of PPAR-α increased significantly under a *GP* treatment [37]. Although there is no direct evidence to prove that *GP* can reduce the TC and TG levels of laying hens by affecting the PPAR pathway, our sequencing results showed that *GP* can affect the expression of the key genes in the PPAR pathway. Moreover, according to the aforementioned studies in other animals, *GP* may affect the serum TC and TG levels by regulating the key genes in the PPAR pathway. These data provide new insights into the application of *GP* in the treatment of inflammation and fatty acid metabolism.

Furthermore, *GP*-induced alterations in the expression of genes such as *CYP7A1*, *FABP3*, *SCD1*, and *PCK1* are involved in the lipid metabolism. Cholesterol 7-alpha hydroxylase (*CYP7A1*) is a speed-limiting enzyme that catalyzes the decomposition of cholesterol into bile acids in the liver. Its main function is to maintain the balance of the cholesterol metabolism and is easily affected by food, drugs, and other factors [42]. Ramakrishna et al. found that 16-dehydropregnenolone (DHP) profoundly decreased the serum TC and TG levels and the hypolipidemic activity of DHP by upregulating the hepatic *CYP7A1* pathway that promotes cholesterol-to-bile acid conversion and bile acid excretion [43]. Soy milk and fermented soy milk contain isoflavones; additionally, soy milk reduces the liver cholesterol and triglyceride levels and increases the liver *CYP7A1* gene expression in rats [44]. In our quantitative analysis, we found that the addition of *GP* significantly upregulated *CYP7A1*. The effect of *GP* on the triglycerides and cholesterol of the layer serum may be due to the upregulation of *CYP7A1* expression by flavonoids. The fatty acid-binding protein (FABP) mediates the anabolism and catabolism of the lipid metabolism pathway, maintains the level of fatty acids in cells [45], and regulates the transcription of fatty acid-related genes [46]. Wang et al. found that the expression level of *FABP3* was positively correlated with the levels of the cholesterol and triglycerides. Interestingly, the expression levels of FABP1, FABP3, and FABP10 in hens during the peak laying period were significantly higher than those before laying [47]. Stearoyl-CoA desaturase 1 (*SCD1*) is a fatty acid desaturase. After the knockout of *SCD1*, the contents of TG, TC, and the desaturase index decreased significantly. The deletion of *SCD1* decreased the expression of other genes involved in de novo fatty acid synthesis, including *FABP3* and *FABP4* [48]. *SCD1* inhibition in cardiomyocytes decreased the levels of lipogenic proteins and increased lipolysis. Moreover, the downregulation of hepatic *SCD1* expression caused a marked decrease in the phosphoenolpyruvate carboxykinase (PEPCK, encoded by the *PCK1* gene) expression in rodents. In this study, we found that the addition of *GP* to laying hen feed reduced the expression of *SCD1*, *PCK1*, and *FABP3* in the liver. The addition of *GP* may lead to the downregulation of *SCD1*, which affects the decrease of fatty acid synthesis-related genes such as FABP3, and the downregulation of the main control point for the regulation of gluconeogenesis, PCK1, thus affecting the synthesis of TG and TC. There are few studies on ankyrin repeat domain 22 (*ANKRD22*) and related studies in medicine that have shown that the expression levels of *ANKRD22* in human breast cancer [49] and pancreatic cancer [50] tissues are significantly higher than those in normal breast tissues. In colorectal cancer-initiating cells (CCICs), ANKRD22 cooperates with the lipid transport protein extended synaptotagmin-1 (ESYT1) to transport excess lipids into the mitochondria and reduce the number of mitochondria in an autophagy-independent manner, thus meeting the metabolic requirements of CCICs [51]. These results indicate that ANKRD22 is also associated with lipid metabolism. 

## 5. Conclusions

In conclusion, *Gynostemma pentaphyllum* (*GP*) has a lipid-lowering effect on the liver by regulating the key transcriptional factors and lipogenic enzymes involved in fatty acid oxidation during hepatic lipogenesis. The present study proves that the addition of *GP* to layer feed can reduce the serum and yolk cholesterol. This effect could potentially be attributed to the addition of *GP*, which affects the expression of key genes in the PPAR-signaling pathway. At the same time, *GP* may also have anti-inflammatory effects on laying hens by inhibiting the secretion of triglycerides by the liver. The present study also demonstrated a potential new effect of *GP* on the yolk cholesterol and autoimmunity in laying hens. In addition, the RNA-seq of liver tissues in hens suggests that their immunity and fatty acid oxidation should be further evaluated. Given these positive results, favorable chronic toxicological evaluations in poultry clinical trials have been initiated.

## Figures and Tables

**Table 1:** Nutritional value and active compounds of *Gynostemma pentaphyllum* [23].

Nutritional Composition	Content	Active Compounds	Content
Carbohydrate	7 g	Saponin	2–10%
Protein	4.7 g	Polysaccharide	3–10%
Fat	0.3 g	Flavonoids	3–5%
Dietary fiber	32 g		
Vitamin B1	0.09 mg	Saponins have the effects of lowering blood lipid, sedation, and hypnosis	
Vitamin B2	0.27 mg	Polysaccharides can regulate immunity, reduce blood sugar, and have antioxidation and antitumor effects	
Vitamin C	12 mg	Flavonoids and saponins have antioxidant and antitumor effects together	
Vitamin E	46 mg		
Vitamin A	2.95 mg		
Threonine	0.1425 mg		
Methionine	0.3289 mg		
Leucine	0.0549 mg		
Isoleucine	0.2127 mg		
Phenylalanine	0.9758 mg		
Lysine	1.5563 mg		
Niacin	1.1 mg		

**Table 2:** The composition of the basal diet (as-fed basis).

Composition	Growth Stage			
	0–8 Weeks	9–14 Weeks	15 Weeks—Age at First Eggs	Age at First Eggs—60 Weeks
Corn (%)	62.5	61.5	60.0	62.5
Wheat bran (%)	4.0	13.0	19.5	-
Soybean meal (%)	28.0	20.0	15.0	24.0
Soybean oil (%)	0.5	0.5	0.5	0.5
Coarse stone powder (%)	-	-	-	8
Premix ^1^ (%)	5	5	5	5
Total (%)	100	100	100	100
Dry matter content (%)	87.51	87.36	87.27	88.31
Crude protein (%)	18.31	16.26	15.03	15.93
Calcium (%)	0.87	0.86	0.85	3.83
Total phosphorus (%)	0.59	0.62	0.65	0.52
Available phosphorus (%)	0.35	0.35	0.36	0.32
Salt (%)	0.36	0.37	0.38	0.35
Lysine (%)	0.98	0.85	0.76	0.86
Methionine (%)	0.41	0.36	0.34	0.39

^1^ Provided the following per kilogram of premix: Fe (Fe_2_(SO_4_)_3_)100 g, Cu (CuSO_4_) 8 mg, Mn (MnSO_4_) 80 mg, I (Ca(IO_3_)2) 0.5 mg, Se (Na_2_SeO_3_ ) 0.2 mg, VA 12,000 IU, VD3 2200 IU, VE 30 IU, VB1 2 mg, VB2 5 mg, VB6 1.5 mg, VB12 0.03 mg, folic acid 1.25 mg, nicotinic acid 40 mg, D-pantothenic acid 10 mg, and biotin 0.36 mg.

**Table 3:** Effects of dietary-supplemented *Gynostemma pentaphyllum* on the growth performance of laying hens at 2–20 weeks.

Week	Groups			SEM	*p*-Value
	Control Group	LA Group	HA Group		
Initial weight (g)	75.40	73.99	74.50	1.61	0.4390
Final weight (g)	1376.19	1342.00	1394.21	25.15	0.6511
ADG (g/d)	10.32	10.06	10.47	0.47	0.5845
ADFI (g/d)	52.87	50.01	51.76	1.21	0.4397
FCR	5.12	4.97	4.94	0.11	0.5173

Data are expressed as the mean and SEM (standard error of the mean, *n* = 5). LA group = low-addition group, HA group = high-addition group, ADFI = average daily feed intake, ADG = average daily gain, and FCR = feed conversion ratio. No superscripts mean insignificant differences (*p* > 0.05).

**Table 4:** Effects of dietary-supplemented *Gynostemma pentaphyllum* on the physical indexes of the eggs.

Project	Groups			SEM	*p*-Value
	Control Group	LA Group	HA Group		
ESI	1.29	1.28	1.29	0.03	0.0974
EW(g)	53.87 ^B^	55.82 ^A^	55.23 ^A^	0.55	0.0100
EST of blunt end (mm)	0.40	0.41	0.42	0.02	0.3086
EST of middle part (mm)	0.40	0.42	0.41	0.03	0.3511
EST of sharp end (mm)	0.40	0.42	0.42	0.02	0.1298
The average EST (mm)	0.40	0.42	0.42	0.02	0.1254

Data are expressed as the mean and SEM (*n* = 20). LA group = low-addition group, HA group = high-addition group, ESI = egg shape index, EW = egg weight, and EST = eggshell thickness. ^A,B^ indicate the difference is very significant (*p* < 0.01), while no superscripts or the same superscripts mean insignificant differences (*p* > 0.05).

**Table 5:** Effects of dietary-supplemented *Gynostemma pentaphyllum* on the egg quality of laying hens.

Project	Groups			SEM	*p*-Value
	Control Group	LA Group	HA Group		
HU	73.18 ^b^	76.04 ^a^	76.89 ^a^	1.03	0.0421
The cholesterol in the yolk (mmol/L)	3.69 ^A^	4.02 ^A^	1.83 ^B^	0.23	0.0078
EYP (%)	24.93	24.87	25.11	0.31	0.2553
Color of yolk (L*—lightness)	69.33	69.47	69.01	0.29	0.3845
Color of yolk (a*—redness)	16.36 ^B^	16.54 ^B^	17.23 ^A^	0.15	0.0043
Color of yolk (b*—yellowness)	60.87	60.74	61.24	0.64	0.1294

Data are expressed as the mean and SEM (*n* = 20) (*n* = 15 for the cholesterol in the yolk). LA group = low-addition group, HA group = high-addition group, HU = Haugh unit, and EYP = egg yolk proportion. ^A,B^ indicate the difference is very significant (*p* < 0.01), and ^a,b^ indicate the difference is significant *p* < 0.05, while no superscripts or the same superscripts mean insignificant differences (*p* > 0.05).

**Table 6:** Effects of dietary-supplemented *Gynostemma pentaphyllum* on the serum biochemical indexes of laying hens.

Index	Groups			SEM	*p*-Value
	Control Group	LA Group	HA Group		
TC (mmol/L)	2.43 ^A^	1.80 ^B^	1.87 ^B^	0.09	0.0038
TG (mmol/L)	5.72 ^A^	3.38 ^B^	3.56 ^B^	0.35	0.0065
TP (g/L)	40.41	37.78	39.01	0.10	0.0783

Data are expressed as the mean and SEM (standard error of the mean, *n* = 15). LA group = low-addition group, HA group = high-addition group, TC = total cholesterol, TG = triglycerides, and TP = total protein. ^A,B^ indicate the difference is extremely significant (*p* < 0.01), while no superscripts or the same superscripts mean insignificant differences (*p* > 0.05).

## Data Availability

The data concerning this study are available in the article and the Supplementary Materials. The data are available at NCBI SRA: SUB10233136.

## Supplementary Materials

The following are available online at www.mdpi.com/article/10.3390/genes12121942/s1, Table S1: The primer for qRT-PCR validation. Table S2: Quality control results and high-quality clean reads obtained for each sample. Table S3: Differentially expressed genes between EG and CG.
